# Neural Markers of Methylphenidate Response in Children With Attention Deficit Hyperactivity Disorder

**DOI:** 10.3389/fnbeh.2022.887622

**Published:** 2022-05-06

**Authors:** Anne B. Arnett, Tara M. Rutter, Mark A. Stein

**Affiliations:** ^1^Division of Developmental Medicine, Boston Children’s Hospital, Boston, MA, United States; ^2^Department of Pediatrics, Harvard Medical School, Cambridge, MA, United States; ^3^Department of Psychology, Seattle Pacific University, Seattle, WA, United States; ^4^Department of Psychiatry & Behavioral Medicine, Seattle Children’s Hospital, Seattle, WA, United States; ^5^Department of Psychiatry & Behavioral Sciences, University of Washington, Seattle, WA, United States

**Keywords:** ADHD, EEG, ERP, aperiodic slope, methylphenidate, precision medicine

## Abstract

**Background:**

Despite widespread use of stimulants to treat ADHD, individual responses vary considerably and few predictors of response have been identified. The identification of reliable and clinically feasible biomarkers would facilitate a precision medicine approach to pharmacological treatment of ADHD. We test the hypothesis that two electroencephalography (EEG) based neural signatures of ADHD, resting aperiodic slope exponent and novelty P3 amplitude, are markers of methylphenidate response in children. We hypothesize that positive response to methylphenidate treatment will be associated with greater abnormality of both neural markers.

**Methods:**

Twenty-nine 7-11 year-old children with ADHD and a history of methylphenidate treatment, and 30 controls completed resting EEG and visual oddball event related potential (ERP) paradigms. ADHD participants were characterized as methylphenidate responders (*n* = 16) or non-responders (*n* = 13) using the clinical global improvement (CGI-I) scale during blinded retrospective interview. All participants abstained from prescribed medications for at least 48 hours prior to the EEG.

**Results:**

As expected, methylphenidate responders (CGI-I rating < 3) demonstrated attenuated P3 amplitude relative to controls. Unexpectedly, methylphenidate non-responders showed atypically flat aperiodic spectral slope relative to controls, while responders did not differ on this measure.

**Conclusion:**

ADHD symptoms associated with atypical patterns of intrinsic neural activity may be less responsive to methylphenidate. In contrast, ADHD symptoms associated with abnormal frontal-striatal neural network excitation may be correctable with methylphenidate. Altogether, EEG is a feasible and promising candidate methodology for identifying biomarkers of stimulant response.

## Introduction

ADHD is the most common neurodevelopmental disorder diagnosed in children. The number of and use of approved medications for ADHD has increased over the past four decades (Steingard et al., 2019). The most commonly prescribed medications include methylphenidate and amphetamine stimulant classes, with the former recommended as the first line of treatment in children and adolescents (Cortese et al., 2018; Cortese, 2020). While stimulant medications can quickly reduce ADHD symptoms and improve behavioral and psychosocial functioning in many children with ADHD, a substantial proportion of affected children do not respond to stimulants (25-35%; Pelham et al., 1999; Stein et al., 2011), and long-term adherence or continuation is poor. Moreover, a significant percentage demonstrate preferential response to one stimulant class over another (∼40%; Arnold, 2000; Stein et al., 2011) and as a result, treatment guidelines suggest trying the alternative stimulant class in cases of non-response (Pliszka and Issues, 2007). Despite increasing interest in precision medicine approaches, as yet no evidence-based guidelines exist to direct providers toward a specific treatment for a particular child (Owens et al., 2003). As a result, a trial-and-error clinical approach has been maintained. This can lead to premature termination of titration trials, lack of dose optimization, patient dissatisfaction, and prolonged functional impairment.

Aperiodic spectral slope, also referred to as the 1/*f* distribution (where *f* is derived from the shape of the power spectral density S[*f*]), neural pink noise, and scale-free dynamics (He et al., 2010; Voytek et al., 2015; Donoghue et al., 2020a), is an index of spontaneous intrinsic brain activity measured with electroencephalography (EEG). Similar to a traditional spectral power distribution, the aperiodic slope can be illustrated as a distribution of oscillatory power across a range of frequencies; however, unlike traditional spectral power distributions, the aperiodic slope captures background activity absent of periodic peaks in a particular frequency range, such as the dominant alpha peak (Donoghue et al., 2020a). The steepness of the aperiodic slope is quantified by the aperiodic exponent (i.e., the *X* in 1/*f^x^*). Greater aperiodic exponent reflects increased excitatory signal and engagement of global neural populations, while flatter slope reflects a shift toward inhibitory currents and local neural networks (Waschke et al., 2019). Thus, aperiodic activity potentially reflects a balance of segregation and integration of neural information across space and time. To this extent, aperiodic slope may be thought of as measure of bottom-up neurocognitive processes that influence basic sensory processing as well as higher order stimulus categorization and cognitive control.

Individual variation in aperiodic spectral slope have been linked to demographic and developmental differences. The aperiodic exponent decreases (i.e., becomes flatter) with age across the lifespan (Cellier et al., 2021; McSweeney et al., 2021; Pathania et al., 2022). Adolescent males have greater aperiodic exponents than females (McSweeney et al., 2021). Among elderly individuals, reduced aperiodic exponent is associated with greater cognitive decline (Pathania et al., 2022). Several studies have reported that children and adolescents with ADHD have reduced aperiodic exponent compared to same age controls (Arnett et al., in press; Ostlund et al., 2021), although the opposite effect may be true for preschool aged children (Robertson et al., 2019). There is emerging evidence that stimulant medication moderates the aperiodic exponent. Pertermann et al. (2019) found that the aperiodic exponents of school aged children with ADHD increased following optimized methylphenidate treatment, and this difference was correlated with improved behavioral performance. Interestingly, two studies in which the participants abstained from medication prior to the EEG also found an effect of medication history on aperiodic slope. Robertson et al. (2019) reported that preschool aged children with a history of stimulant use had relatively normalized aperiodic slope compared to controls. In contrast, our group found that children who were normally medicated for ADHD had more extreme flattening of aperiodic slope, although we hypothesized that this may be due to higher symptom severity in this sample (Arnett et al., in press). Finally, Ostlund et al. (2021) did not find an association between stimulant history and aperiodic exponent. To our knowledge, no study to date has investigated whether aperiodic spectral slope differs between stimulant responders and non-responders.

In contrast to the aperiodic slope, there is a large literature documenting attenuated amplitude of the P300 event related potential (ERP) component among children and adults with ADHD (Barry et al., 2003). In particular, individuals with ADHD show reduced amplitude of an early subcomponent of the P300 when presented with salient or novel stimuli, such as in the context of an oddball paradigm. Oddball tasks vary frequent presentation of a repeated stimulus (standard) with infrequent presentation of slightly different (deviant) and/or highly original (novel) stimuli (Goldstein et al., 2002). This novelty P3a component is generated by excitation of fronto-striatal neural populations involved in auditory or visual stimulus orientation and evaluation (Polich, 2007). Fronto-striatal neural circuitries are modulated by catecholamine neurotransmitters, particularly dopamine. Accordingly, P3a amplitude has been shown to increase to normal levels following administration of stimulant medications (Peisch et al., 2021), which are dopamine agonists. P3a amplitude is reduced in clinical samples with low dopamine levels, such as Parkinson’s Disease (Solís-Vivanco et al., 2015). Therefore, attenuated novelty P3a amplitude in ADHD is thought to reflect abnormal top-down allocation of cognitive resources during stimulus processing possibly *via* reduced integrity of the fronto-striatal cortical networks, and/or reduced excitatory synaptic capacity.

A small body of literature has reported associations among methylphenidate treatment, behavioral improvement, and increased amplitude of the novelty P3a or related components, among children and adolescents with ADHD. For example, Dolu et al. (2019) found decreased ADHD symptom severity and increased novelty P3 amplitude among school-aged children following methylphenidate administration. Similarly, Hermens et al. (2005) reported that methylphenidate was associated with increased P3 amplitude and enhanced task performance during an auditory oddball task with adolescents. Lopez et al. (2004) reported improved Stroop task performance and increased visual novelty P3 amplitude in children following methylphenidate treatment. Very few studies have directly investigated whether ERP components predict behavioral response to methylphenidate or other stimulant medications. Ogrim and colleagues (Ogrim et al., 2014; Ogrim et al., 2016; Ogrim and Kropotov, 2019) have repeatedly found that attenuated pre-treatment P3 amplitude during a cued go-no-go task is predictive of positive behavioral response to methylphenidate or dexamphetamine stimulants. In contrast, Sangal and Sangal (2006) found response to methylphenidate was associated with greater amplitude of the late auditory (but not visual) P300 component with weak sensitivity (65%) and specificity (67%). This group did not find ERP differences associated with response to atomoxetine.

The current study tests the hypothesis that two electrophysiological indices of neurocognitive functioning will be associated with methylphenidate response in children with ADHD. First, we hypothesize that the aperiodic exponent and novelty P3a amplitude are potential biomarkers of individual response to methylphenidate among school age children with ADHD. Secondly, we hypothesize that children with evidence of both bottom-up and top-down cognitive dysregulation (i.e., flat aperiodic slope and attenuated P3a amplitude, respectively) will show greater response to methylphenidate, relative to children with normal neurocognitive functioning in one or both measures.

## Materials and Methods

### Participants

Participants were 29 children, ages 7-11 years, with a confirmed DSM-5 diagnosis of ADHD and a history of methylphenidate treatment, and 30 control children. The sample was recruited from a larger cohort (ADHD *n* = 100; control *n* = 30) who had completed a study of neurocognitive correlates of ADHD at the University of Washington (Rutter and Arnett, 2020; Arnett et al., 2021). The parent study recruited participants with a history of ADHD and non-ADHD controls from local outpatient psychiatric clinics, pediatric primary care practices, distribution of fliers to community partners, and posts to relevant social media sites. ADHD diagnoses were confirmed by a licensed clinical psychologist *via* review of caregiver report on the CBCL 6-18 (Achenbach, 2014) and an ADHD checklist, caregiver report on the K-SADS-COMP (Townsend et al., 2019), clinical interview with the caregiver, and/or behavioral observation during the parent study research visit. Exclusion criteria for both ADHD and control participants were a diagnosis of autism spectrum disorder, intellectual disability, gestational age <32 weeks, genetic disorder, prenatal exposure to alcohol or drugs, or colorblindness. Control children additionally did not have concern for ADHD or an immediate family history of ADHD. Other DSM diagnoses (e.g., anxiety, depression, and learning disorders) were not exclusionary for either group; however, the control group was largely free of psychiatric symptoms.

### Procedures

Informed consent and assent were obtained from participating caregivers and children in compliance with the approved University of Washington IRB protocol (STUDY00004534). Parent study procedures included a single laboratory visit during which the child completed a one-hour EEG and neuropsychological testing. Caregivers reported on their child’s medical, behavioral, psychiatric, and temperament histories through a combination of in-person and remote data collection procedures. All children abstained from stimulant medications for at least 48 h prior to the visit.

All ADHD participants from the parent study were emailed within two years of parent study participation with an invitation to participate in a remote follow-up if their child had ever been treated with methylphenidate. As part of the follow-up, caregivers completed two retrospective reports in which they were instructed to rate their child’s ADHD symptoms (1) before methylphenidate treatment and (2) at the time the child was taking an optimal dose of methylphenidate, using the Strengths and Weaknesses of ADHD and Normative Behavior (SWAN) scale (Swanson et al., 2012). Optimal methylphenidate dose was defined as either (1) the highest dose taken for longer than three days when the medication trial was discontinued before one month, or (2) dose at which the caregiver believed the child demonstrated the best behavioral response, when methylphenidate treatment lasted longer than one month. Caregivers then completed a telephone interview, and a trained clinical investigator rated medication-related severity and improvement using the clinical global impressions severity scale (CGI-S; Guy, 1976), as well as the child’s response to the optimal methylphenidate dose using the clinical global impressions improvement (CGI-I) scale. CGI interviews were completed an average of 23 months (range = 14 – 33 months) after the EEG.

Continuous EEG was acquired with a 128-channel Magstim-EGI Hydrocel geodesic sensor net and Netstation Acquisition software version 4.5.6, integrated with a 400-series high impedance amplifier (Magstim-EGI; Plymouth, MN). Electrode impedances were reduced to below 50 kOhms at the start of the session and monitored throughout. EEG signals were referenced to the vertex electrode, analog filtered (0.1 Hz high-pass, 100 Hz elliptical low-pass), amplified and digitized with a sampling rate of 1,000 Hz. Timing of the presentation of the visual stimuli was recorded using a Cedrus Stimtracker (Cedrus Corporation, San Pedro, CA). Continuous EEG data were subsequently processed offline in MATLAB R2018b using EEGLAB 15 and ERP Lab v8.0 functions. Eye electrodes and 14 rim channels were excluded from analyses. Data were downsampled to 250 hz and bandpass filtered at 0.3-80 hz. Electrical line noise from 55 to 65 hz was removed using the Cleanline plugin for EEGLAB. Following methods outlined in the Harvard Automated Processing Pipeline for Electroencephalography (HAPPE; Gabard-Durnam et al., 2018), channels with normed joint probability more than 3 standard deviations beyond the average log power (in the range of 1-125 Hz) were automatically rejected. This step was performed twice. Subsequently, rejected channels were interpolated back into the dataset prior to average referencing. Extended independent component analysis (ICA) was run with primary component analysis dimension reduction to identify and subsequently remove artifactual independent components (e.g., eye blinks, line noise or cardiac signal), consistent with published pipelines (Levin et al., 2018).

### Measures

#### Methylphenidate Response

CGI-I ratings were used to characterize ADHD participants as methylphenidate responders or non-responders. Consistent with previous literature indicating that CGI-I ratings of “much improved” or “very much improved” correspond to a 30% reduction in symptoms (Mattingly et al., 2017), CGI-I scores below three were characterized as methylphenidate responders, while those with CGI-I scores greater than or equal to three (“minimally improved,” “no change,” “minimally worse,” “much worse,” or “very much worse”) were characterized as non-responders. Sixty-nine percent (*n* = 11) of the responders were currently taking a methylphenidate medication at the time of the interview, as opposed to 31% (*n* = 4) of the non-responders, further supporting our characterization approach.

#### Aperiodic Exponent

Aperiodic slope exponents were acquired from a lights-off resting EEG paradigm. During the resting EEG, participants were seated in a comfortable chair in a dark room and instructed to sit quietly with their eyes open for two minutes. The lights off condition was selected over an “eyes-closed” resting condition to minimize muscle artifact and potential cognitive effort that is often introduced by asking young children to keep their eyes closed. After processing, the amount of usable data ranged from 85 to 120 s.

Aperiodic exponents were computed in MATLAB. Welch’s method was used to perform fast Fourier transformation (FFT) on resting EEG data with a 50% overlap and 1-second Hamming window. Next, the Fitting Oscillations and One-Over-f (FOOOF) MATLAB toolbox (Donoghue et al., 2020b) was used to compute the aperiodic exponent across a frequency range of 1-50 Hz, at each electrode. Consistent with prior literature (Robertson et al., 2019; Ostlund et al., 2021), we specified a fixed aperiodic slope calculation after visual inspection did not indicate a “knee” in individual power spectral density distributions. Other parameters were specified as follows: *peak_width_limits* = [2,12], *max_n_peaks* = 8, *min_peak_height* = 0.5, *peak_threshold* = 2.0. For each individual, a mean aperiodic exponent was calculated as the average exponent across electrodes in five midline scalp regions: anterior frontal (Afz, Af3, Af4), frontal (Fz, F3, F4), central (Cz, C3, C4), parietal (Pz, P3, P4) and occipital (Oz, O3, O4).

#### Novelty P3a

The ERP task was completed following the resting EEG. One-hundred-forty target (task-related) visual stimuli were presented alternately with 140 oddball (non-task-related) visual stimuli, using a design adapted from Jonkman et al. (1997). Targets were red, blue, green, and orange rectangles. Oddball stimuli were a white bracket presented 60% of the time; an identical bracket oriented in the opposite direction presented 20% of the time; and non-repeated white line drawings of animals and vehicles, presented 20% of the time (see Supplementary Figure 1). The latter stimuli were considered “novel” and were the focus of the current analyses (see Supplementary Figure 2 for a grand average waveform depicting amplitude differences across each condition). All stimuli were presented against a black background, with a duration of 300 ms and interstimulus interval of 0.8-1.4 seconds. During the ERP task, participants were given instructions to respond to target stimuli consistent with a traditional forced-choice discrimination task (right-hand button press to blue rectangles and left-hand button press to all other colors). Participants were instructed to “ignore” the oddball stimuli, which were presented between each target. Participant behavior was monitored by the experimenter *via* camera and “bad trials” in which the child was not attending to the task or moving excessively were coded for exclusion from the analyses. Task accuracy did not differ between ADHD and control groups (*t*[55.01] = −0.46, *p* = 0.650).

**FIGURE 1:**
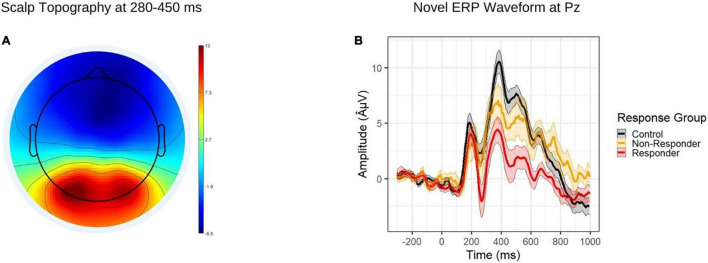
**(A)** Topographic map depicting grand average amplitudes to novel stimuli during the P3a time window (280-450 ms). **(B)** ERP waveform to novel stimuli at the Pz electrode, by response group. The shaded regions depict standard errors of the mean.

Event related potential task data were segmented to 300 milliseconds prior and 900 milliseconds following presentation of novel stimuli on the participant screen. Epochs were further lowpass filtered at 40 Hz and baseline corrected using the full 300 ms. Based on visual inspection of ERP waveforms, average amplitude of the novelty P3a component was extracted from the Pz electrode during the 280-450 ms window (Figure 1A). After processing, the number of novel stimulus trials (out of a possible 28) was comparable across diagnostic groups (*t*[54.15] = −0.37, *p* = 0.714; ADHD range = 22-28; control range = 16-28).

#### Analytic Plan

Data were prepared and analyzed in R Studio version 2021.09.1. Continuous variables (i.e., aperiodic exponent and P3a amplitude) were normally distributed. To address our main hypothesis, we tested a multinomial logistic regression with methylphenidate response group as the unordered categorical dependent variable, and controls specified as the reference group. Independent variables were race, aperiodic exponent, and novelty P3a amplitude. Next, we examined the ability of our model to distinguish between responders and non-responders using receiver operating characteristic (ROC) analysis (R package pROC). The ADHD sample was randomly split into 200 test (70%) and train (30%) datasets. Area under the curve (AUC) and 95% confidence intervals (CI) were calculated for each set, along with sensitivity and specificity estimates at five thresholds. CI estimates were bootstrapped over 2,000 stratifications for each calculation.

## Results

Sample demographics are described in Table 1. ADHD and control subjects did not differ on age, proportion of non-White participants, or proportion of females; however, the control group had a significantly higher abbreviated IQ than the ADHD group, as measured with the two-subtest version of the Wechsler Abbreviated Scale of Intelligence, 2nd Edition (WASI-II) (Wechsler, 2011). Demographic differences across the methylphenidate response groups (controls, responders and non-responders) were also examined. Responders were more likely to be described as white by their caregivers (χ^2^[2] = 6.51, *p* = 0.039). Response groups did not differ on age (*F*[2,52] = 1.48, *p* = 0.236) or proportion of females (χ^2^[2] = 1.54, *p* = 0.462). Responders and non-responders did not differ on IQ (*p* = 0.967). Thus, only the binomial self-identified race variable (white versus non-white) was included in subsequent analyses.

**TABLE 1:** Sample demographics.

	ADHD	Control	*p*
*n*	29	30	
Age in months (SD)	121.08 (16.90)	114.39 (15.85)	0.123
Female	28%	33%	0.844
Non-White	31%	33%	1.000
Abbreviated IQ	108.52 (9.87)	117.50 (10.45)	**0.001**

Results of the multinomial logistic regression model are reported in Table 2. As expected, methylphenidate responders showed attenuated P3a amplitude relative to controls (Figure 1B), while non-responders did not.

**TABLE 2:** Multinomial logistic regression predicting methylphenidate response.

	Responder	Non-responder
Race	2.28 (1.03), *p* = 0.027	−0.56 (0.79), *p* = 0.480
Aperiodic Exponent	−4.28 (2.41), *p* = 0.076	−5.47 (2.60), *p* = 0.036
Novelty P3a Amplitude	−0.31 (0.11), *p* = 0.005	−0.09 (0.08), *p* = 0.294

*Reference group = control. Values are unstandardized coefficients, with standard errors in parentheses. Race variable dummy coded as 0 = non-white, 1 = white.*

**FIGURE 2:**
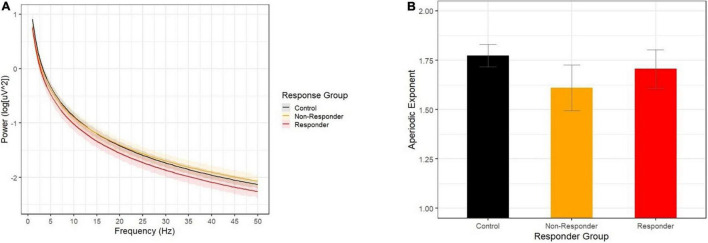
**(A)** Aperiodic slopes by response group show that the non-responder group (yellow) showed a flatter decline across 1-50 Hz than did the control group (black), while the responder group (red) did not differ from controls. **(B)** Aperiodic slope exponents by response group.

Contrary to our predictions, the opposite pattern emerged for aperiodic slope (Figure 2). Methylphenidate non-responders had flatter aperiodic slope than did controls, while the aperiodic exponent did not differ between responders and controls (although the effect approached significance in the expected direction). Finally, children described as white by their caregivers were more likely to be characterized as methylphenidate responders.

Finally, we examined the ability for the combined P3a and aperiodic slope indices to predict responder group membership. The average AUC was 0.79 (95% CI: 0.50 – 0.99), with an optimized sensitivity/specificity of 0.71 and 0.70, respectively.

## Discussion

Aperiodic spectral slope and novelty P3a amplitude measure distinct neural processes, with the former reflecting global patterns of intrinsic activity at rest and the latter representing time-locked activation of fronto-striatal neuronal populations. In the current study, the combination of these measures achieved moderate to high accuracy for predicting retrospective report of response to methylphenidate in a small sample of children with ADHD. More specifically, methylphenidate responders and non-responders showed opposing patterns of neural atypicality, wherein responders were characterized by attenuated ERP amplitude and non-responders characterized by reduced aperiodic exponent.

These preliminary results indicate that methylphenidate, which increases catecholamine availability in the synapse, may be more effective for children whose blunted P3a amplitudes suggest reduced excitation of neuronal populations in the frontal-striatal cortices. This is consistent with a substantial body of research demonstrating that methylphenidate enhances (i.e., “normalizes”) P3a amplitude among children with ADHD (Peisch et al., 2021). The implication is that increased availability of catecholamine neurotransmitters normalizes the functioning of the fronto-striatal networks, possibly by facilitating excitation of larger neural populations.

In contrast, our results suggest that methylphenidate may be less effective for children with intact P3a amplitudes and flatter aperiodic slope. The aperiodic slope is a measure of the balance of slower versus faster background neural oscillations. While slower oscillations are generated by larger, coordinated global neuronal populations, faster oscillations generally reflect activity in smaller, local neural networks. Thus, the balance of slow and fast aperiodic oscillations represents an intrinsic “self-tuning” by the brain that supports flexible and efficient neuronal activation in response to periodic environmental elements. In a dark room at rest, as in the current study, the brain is expected to show a greater emphasis toward engagement of long-range, global networks (i.e., steeper aperiodic slope), while in an environment with more visual or auditory input, the emphasis shifts toward involvement of smaller, more segregated networks (i.e., flatter aperiodic spectral slope). Dynamic shifts of this balance in response to environmental conditions, which have previously been demonstrated to be atypical in ADHD populations (Arnett et al., 2021), facilitate efficient processing of environmental input.

If flatter aperiodic slope is a mechanism contributing to ADHD symptoms among non-responders, then our study may indicate that increased availability of catecholamines is not as effective at normalizing intrinsic activity patterns in the brain. However, results of a prior study were inconsistent with this theory, in that they found methylphenidate treatment did indeed increase the aperiodic exponent in children with ADHD (Pertermann et al., 2019). Methodological differences may explain these divergent conclusions. First, Pertermann et al. (2019) used a method of calculating the aperiodic slope that Ostlund et al. (2021) have reported results in different values than those produced by FOOOF (the method used in the current study). Second, the aperiodic slope calculated by Pertermann et al. (2019) was measured while children were performing a go-no-go task, which in healthy brains, would be expected to lead to flattening of the aperiodic slope relative to our lights-off condition.

Our findings are consistent with prior studies reporting that faster frontal alpha peak frequency in male children and adolescents with ADHD is associated with a history of improved behavioral response to MPH (Arns, 2012; Voetterl et al., 2022). The alpha peak is a periodic feature of the power spectral density distribution generated by the thalamus (Hughes and Crunelli, 2005). Thus, like the P3a, alpha bursts reflect time limited neuronal spiking rather than background oscillatory activity. In future work, it will be important to evaluate the predictive power of multiple periodic and aperiodic signals using a prospective study design.

The current results must be interpreted with caution, however, given the small sample size and retrospective nature of our methylphenidate responder categorization. Prospective studies involving treatment naïve children and consistent optimization of methylphenidate dosing will be necessary to validate our findings. Moreover, while we hypothesize that behavioral effects of methylphenidate are correlated with change in our neurobiological markers, we were not able to re-test these children while on medications as part of the current study. Thus, we cannot confirm that these neural markers of treatment response represent a neurobiological mechanism for the individual child’s ADHD symptoms. Moreover, although all children abstained from prescribed ADHD medications for 48 hours (or longer, for non-stimulants) prior to the EEG, we cannot rule out the possibility that prior stimulant use moderated these neural signatures, a finding that has been suggested in previous work (Pertermann et al., 2019; Robertson et al., 2019). Finally, our analyses were not adequately powered to investigate the potential contribution of co-existing symptomology, such as internalizing and externalizing symptoms. Possibly, inclusion of multimethod indicators (e.g., psychiatric, cognitive, and neural) would improve the predictive accuracy of these models in future research.

Measurement of brain activity using electroencephalography is feasible and thus presents as a strong candidate technique for measuring individual indicators of treatment response. The results of our preliminary work indicate at least two distinct electrophysiological markers of ADHD symptoms were associated with divergent responses to methylphenidate. These findings have potential to improve delivery of precision medicine care for children with ADHD, as well as inform our understanding of the neurobiological mechanisms contributing to the broader ADHD phenotype.

## Data Availability Statement

The raw data supporting the conclusions of this article will be made available by the authors, without undue reservation.

## Ethics Statement

The studies involving human participants were reviewed and approved by University of Washington Institutional Review Board. Written informed consent to participate in this study was provided by the participants’ legal guardian/next of kin.

## Author Contributions

AA wrote the first draft of the manuscript, contributed to study design, conceptualization and data collection, and ran statistical analyses. TR contributed to conceptualization and data collection and reviewed the final manuscript. MS contributed to study design and conceptualization, contributed to manuscript drafts, and reviewed the final manuscript. All authors contributed to the article and approved the submitted version.

## Conflict of Interest

The authors declare that the research was conducted in the absence of any commercial or financial relationships that could be construed as a potential conflict of interest.

## Publisher’s Note

All claims expressed in this article are solely those of the authors and do not necessarily represent those of their affiliated organizations, or those of the publisher, the editors and the reviewers. Any product that may be evaluated in this article, or claim that may be made by its manufacturer, is not guaranteed or endorsed by the publisher.

## Supplementary Material

Supplementary Figure 1 Schematic of the ERP task.

Supplementary Figure 2 ERP waveform for standard, deviant and novel visual oddball stimuli.

## References

[B1] Achenbach T. M. (2014). Achenbach system of empirically based assessment (ASEBA). *Encycl. Clin. Psychol.* 9 13–23. 10.21500/19002386.1810

[B2] Arnett A. B. Fearey M. Peisch V. Levin A. R. (in press). Absence of dynamic oscillatory response to environmental conditions marks attention deficit hyperactivity disorder. *J. Child Psychol. Psychiatry*10.1111/jcpp.13645 PMC9691533 35620850

[B3] Arnett A. B. Rhoads C. Rutter T. M. (2021). Reduced Error Recognition Explains Post-Error Slowing Differences among Children with Attention Deficit Hyperactivity Disorder. *J.Int. Neuropsychol. Soc.* 7 1–11. 10.1017/S1355617721001065 34488920 PMC8935138

[B4] Arnold L. E. (2000). Methyiphenidate vs. amphetamine: Comparative review. *J. Attent. Dis.* 3 200–211. 10.1177/108705470000300403

[B5] Arns M. (2012). EEG-based personalized medicine in ADHD: Individual alpha peak frequency as an endophenotype associated with nonresponse. *J. Neurother.* 16 123–141. 10.1080/10874208.2012.677664

[B6] Barry R. J. Johnstone S. J. Clarke A. R. (2003). A review of electrophysiology in attention-deficit/hyperactivity disorder: II. Event-related potentials. *Clin. Neurophysiol.* 114 184–198. 10.1016/s1388-2457(02)00363-2 12559225

[B7] Cellier D. Riddle J. Petersen I. Hwang K. (2021). The development of theta and alpha neural oscillations from ages 3 to 24 years. *Dev. Cogn. Neurosci.* 50:100969. 10.1016/j.dcn.2021.100969 34174512 PMC8249779

[B8] Cortese S. (2020). Pharmacologic Treatment of Attention Deficit-Hyperactivity Disorder. *N Engl. J. Med.* 383 1050–1056. 32905677 10.1056/NEJMra1917069

[B9] Cortese S. Adamo N. Del Giovane C. Mohr-Jensen C. Hayes A. J. Carucci S. (2018). Comparative efficacy and tolerability of medications for attention-deficit hyperactivity disorder in children, adolescents, and adults: a systematic review and network meta-analysis. *Lancet Psychiatry* 5 727–738. 10.1016/S2215-0366(18)30269-4 30097390 PMC6109107

[B10] Dolu N. Altınkaynak M. Güven A. Özmen S. Demirci E. ízzetoğlu M. (2019). Effects of methylphenidate treatment in children with ADHD: a multimodal EEG/fNIRS approach. *Psychiatry Clin. Psychopharmacol.* 29 285–292. 10.1080/24750573.2018.1542779

[B11] Donoghue T. Dominguez J. Voytek B. (2020a). Electrophysiological frequency band ratio measures conflate periodic and aperiodic neural activity. *eNeuro* 7.ENEURO.0192-20.2020 10.1523/ENEURO.0192-20.2020 32978216 PMC7768281

[B12] Donoghue T. Haller M. Peterson E. J. Varma P. Sebastian P. Gao R. (2020b). Parameterizing neural power spectra into periodic and aperiodic components. *Nat. Neurosci.* 23 1655–1665. 10.1038/s41593-020-00744-x 33230329 PMC8106550

[B13] Gabard-Durnam L. J. Mendez Leal A. S. Wilkinson C. L. Levin A. R. (2018). The Harvard Automated Processing Pipeline for Electroencephalography (HAPPE): standardized processing software for developmental and high-artifact data. *Front. Neurosci.* 12:97. 10.3389/fnins.2018.00097 29535597 PMC5835235

[B14] Goldstein A. Spencer K. M. Donchin E. (2002). The influence of stimulus deviance and novelty on the P300 and novelty P3. *Psychophysiology* 39 781–790. 10.1111/1469-8986.3960781 12462506

[B15] Guy W. (1976). *Clinical Global Impressions, ECDEU Assessment Manual for Psychopharmacology, revised (DHEW Publ. No. ADM 76-338)*. Rockville: National Institute of Mental Health, 218–222.

[B16] He B. J. Zempel J. M. Snyder A. Z. Raichle M. E. (2010). The temporal structures and functional significance of scale-free brain activity. *Neuron* 66 353–369. 10.1016/j.neuron.2010.04.020 20471349 PMC2878725

[B17] Hermens D. F. Williams L. M. Clarke S. Kohn M. Cooper N. Gordon E. (2005). Responses to methylphenidate in adolescent AD/HD: evidence from concurrently recorded autonomic (EDA) and central (EEG and ERP) measures. *Int. J. Psychophysiol.* 58 21–33. 10.1016/j.ijpsycho.2005.03.006 15936104

[B18] Hughes S. W. Crunelli V. (2005). Thalamic mechanisms of EEG alpha rhythms and their pathological implications. *Neuroscientist* 11 357–372. 10.1177/1073858405277450 16061522

[B19] Jonkman L. M. Kemner C. Verbaten M. N. Koelega H. S. Camfferman G. vd Gaag R.-J. (1997). Event-related potentials and performance of attention-deficit hyperactivity disorder: children and normal controls in auditory and visual selective attention tasks. *Biol. Psychiatry* 41 595–611. 10.1016/s0006-3223(96)00073-x 9046992

[B20] Levin A. R. Méndez Leal A. S. Gabard-Durnam L. J. O’Leary H. M. (2018). BEAPP: the batch electroencephalography automated processing platform. *Front. Neurosci.* 12:513. 10.3389/fnins.2018.00513 30131667 PMC6090769

[B21] Lopez J. Lopez V. Rojas D. Carrasco X. Rothhammer P. García R. (2004). Effect of psychostimulants on distinct attentional parameters in attentional deficit/hyperactivity disorder. *Biol. Res.* 37 461–468. 10.4067/s0716-97602004000300010 15515970

[B22] Mattingly G. Childress A. Nordbrock E. Adjei A. Kupper R. Weiss M. (2017). “Clinical response and symptomatic remission with Aptensio XR (methylphenidate extended-release capsules) in children and adolescents with ADHD,” in *Paper presented at the American Professional Society of ADHD and Related Disorders (APSARD) Annual Meeting*, APSARD: Washington, DC. 10.3390/jcm8040461

[B23] McSweeney M. Morales S. Valadez E. A. Buzzell G. A. Fox N. A. (2021). Longitudinal age-and sex-related change in background aperiodic activity during early adolescence. *Dev. Cogn. Neurosci.* 52:101035. 10.1016/j.dcn.2021.101035 34781249 PMC8605214

[B24] Ogrim G. Aasen I. E. Brunner J. F. (2016). Single-dose effects on the P3no-go ERP component predict clinical response to stimulants in pediatric ADHD. *Clin. Neurophysiol.* 127 3277–3287. 10.1016/j.clinph.2016.07.011 27567447

[B25] Ogrim G. Kropotov J. Brunner J. F. Candrian G. Sandvik L. Hestad K. A. (2014). Predicting the clinical outcome of stimulant medication in pediatric attention-deficit/hyperactivity disorder: data from quantitative electroencephalography, event-related potentials, and a go/no-go test. *Neuropsychiatric Dis. Treat.* 10 231–242. 10.2147/NDT.S56600 24523588 PMC3921081

[B26] Ogrim G. Kropotov J. D. (2019). Predicting clinical gains and side effects of stimulant medication in pediatric attention-deficit/hyperactivity disorder by combining measures from qEEG and ERPs in a Cued GO/NOGO task. *Clin. EEG Neurosci* 50 34–43. 10.1177/1550059418782328 29940782 PMC6291902

[B27] Ostlund B. D. Alperin B. R. Drew T. Karalunas S. L. (2021). Behavioral and cognitive correlates of the aperiodic (1/f-like) exponent of the EEG power spectrum in adolescents with and without ADHD. *Dev. Cogn. Neurosci.* 48:100931. 10.1016/j.dcn.2021.100931 33535138 PMC7856425

[B28] Owens E. B. Hinshaw S. P. Kraemer H. C. Arnold L. E. Abikoff H. B. Cantwell D. P. (2003). Which treatment for whom for ADHD? Moderators of treatment response in the MTA. *J. Consul. Clin. Psychol.* 71:540. 10.1037/0022-006x.71.3.540 12795577

[B29] Pathania A. Euler M. Clark M. Cowan R. Duff K. Lohse K. (2022). Resting EEG spectral slopes are associated with age-related differences in information processing speed. *Biol. Psychol.* 168:108261. 10.1016/j.biopsycho.2022.108261 34999166

[B30] Peisch V. Rutter T. Wilkinson C. L. Arnett A. B. (2021). Sensory processing and P300 event-related potential correlates of stimulant response in children with attention-deficit/hyperactivity disorder: A critical review. *Clin. Neurophysiol.* 132 953–966 10.1016/j.clinph.2021.01.015 33677205 PMC7981253

[B31] Pelham W. E. Aronoff H. R. Midlam J. K. Shapiro C. J. Gnagy E. M. Chronis A. M. (1999). A comparison of Ritalin and Adderall: efficacy and time-course in children with attention-deficit/hyperactivity disorder. *Pediatrics* 103 e43–e43. 10.1542/peds.103.4.e43 10103335

[B32] Pertermann M. Bluschke A. Roessner V. Beste C. (2019). The modulation of neural noise underlies the effectiveness of methylphenidate treatment in attention-deficit/hyperactivity disorder. *Biol. Psychiatr.* 4 743–750. 10.1016/j.bpsc.2019.03.011 31103546

[B33] Pliszka S. Issues A. W. G. O. Q. (2007). Practice parameter for the assessment and treatment of children and adolescents with attention-deficit/hyperactivity disorder. *J.Am. Acad. Child Adol. Psychiatr.* 46 894–921. 10.1097/chi.0b013e318054e724 17581453

[B34] Polich J. (2007). Updating P300: an integrative theory of P3a and P3b. *Clin. Neurophys Iol.* 118 2128–2148. 10.1016/j.clinph.2007.04.019 17573239 PMC2715154

[B35] Robertson M. M. Furlong S. Voytek B. Donoghue T. Boettiger C. A. Sheridan M. A. (2019). EEG power spectral slope differs by ADHD status and stimulant medication exposure in early childhood. *J. Neurophysiol.* 122 2427–2437. 10.1152/jn.00388.2019 31619109 PMC6966317

[B36] Rutter T. M. Arnett A. B. (2020). Temperament Traits Mark Liability for Coexisting Psychiatric Symptoms in Children With Elevated ADHD Symptoms. *J. Attention Dis.* 25 1871–1880. 10.1177/1087054720943282 32697164 PMC7931648

[B37] Sangal R. B. Sangal J. M. (2006). Attention-deficit/hyperactivity disorder: use of cognitive evoked potential (P300) to predict treatment response. *Clin. Neurophysiol.* 117 1996–2006. 10.1016/j.clinph.2006.06.004 16890481

[B38] Solís-Vivanco R. Rodríguez-Violante M. Rodríguez-Agudelo Y. Schilmann A. Rodríguez-Ortiz U. Ricardo-Garcell J. (2015). The P3a wave: a reliable neurophysiological measure of Parkinson’s disease duration and severity. *Clin. Neurophysiol.* 126 2142–2149. 10.1016/j.clinph.2014.12.024 25655938

[B39] Stein M. A. Waldman I. D. Charney E. Aryal S. Sable C. Gruber R. (2011). Dose effects and comparative effectiveness of extended release dexmethylphenidate and mixed amphetamine salts. *J. Child Adolescent Psychopharmacol.* 21 581–588. 10.1089/cap.2011.0018 22136094 PMC3243461

[B40] Steingard R. Taskiran S. Connor D. F. Markowitz J. S. Stein M. A. (2019). New formulations of stimulants: an update for clinicians. *J. Child Adolescent Psychopharmacol.* 29 324–339. 10.1089/cap.2019.0043 31038360 PMC7207053

[B41] Swanson J. M. Schuck S. Porter M. M. Carlson C. Hartman C. A. Sergeant J. A. (2012). Categorical and dimensional definitions and evaluations of symptoms of ADHD: history of the SNAP and the SWAN rating scales. *Int. J. Educ. Psychol. Assessment* 10:51. 26504617 PMC4618695

[B42] Townsend L. Kobak K. Kearney C. Milham M. Andreotti C. Escalera J. (2019). Development of Three Web-Based Computerized Versions of the Kiddie Schedule for Affective Disorders and Schizophrenia Child Psychiatric Diagnostic Interview: Preliminary Validity Data. *J. Am. Acad. Child Adol Psych.* 59 309–325 10.1016/j.jaac.2019.05.009 31108163

[B43] Voetterl H. van Wingen G. Michelini G. Griffiths K. R. Gordon E. DeBeus R. (2022). Brainmarker-I differentially predicts remission to various attention-deficit/hyperactivity disorder treatments: a blinded discovery, transfer and validation study. *Biol. Psychiatr.* 28:S2451-9022(22)00046-5 10.1016/j.bpsc.2022.02.007 35240343

[B44] Voytek B. Kramer M. A. Case J. Lepage K. Q. Tempesta Z. R. Knight R. T. (2015). Age-related changes in 1/f neural electrophysiological noise. *J. Neurosci.* 35 13257–13265. 10.1523/JNEUROSCI.2332-14.2015 26400953 PMC4579381

[B45] Waschke L. Donoghue T. Voytek B. Obleser J. (2019). Aperiodic EEG activity tracks 1/f stimulus characteristics and the allocation of cognitive resources. *2019 Conference on Cognitive Computational Neuroscience* (Berlin)

[B46] Wechsler D. (2011). *Wechsler Abbreviated Scale of Intelligence*, 2nd Edn. Bloomington, MN: Pearson.

