# Supplementary figures and images for: Neural Markers of Methylphenidate Response in Children With Attention Deficit Hyperactivity Disorder
Source: Front Behav Neurosci. 2022 May 6;16:887622. doi: 10.3389/fnbeh.2022.887622 (PMC9121006; doi:10.3389/fnbeh.2022.887622)

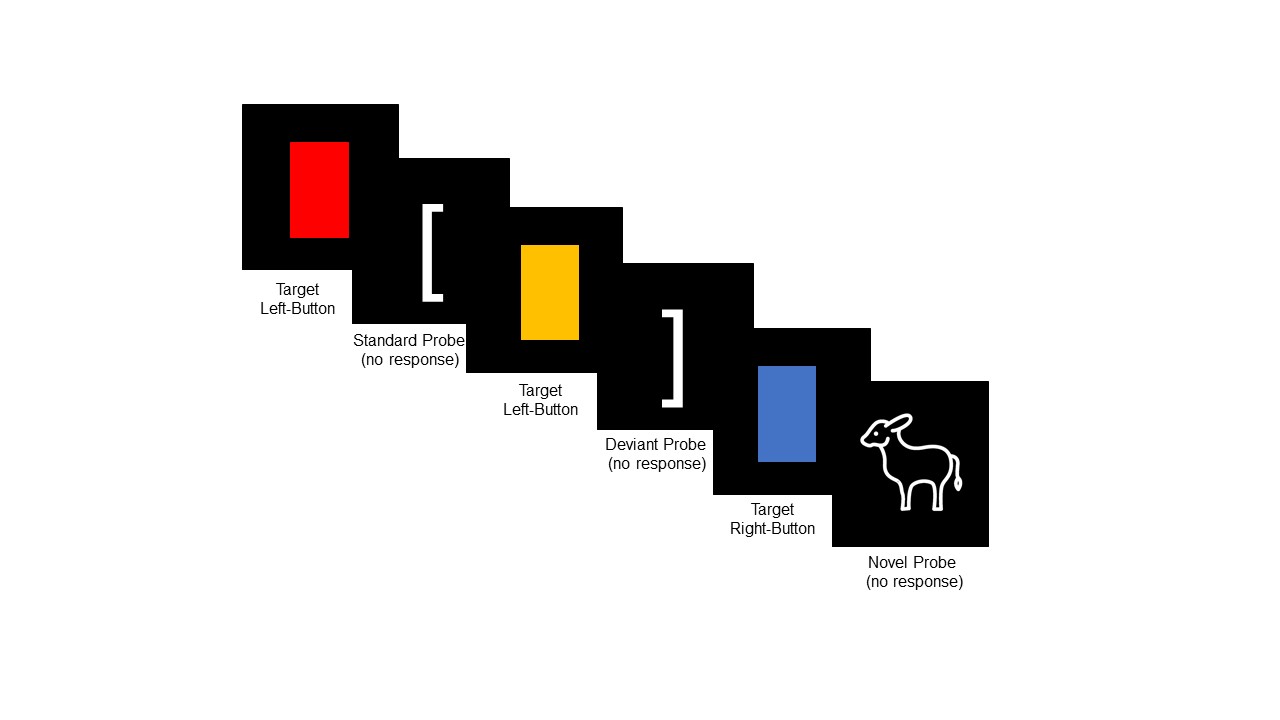

Supplement: Supplementary Figure 1 — Schematic of the ERP task. [file Image_1.JPEG]

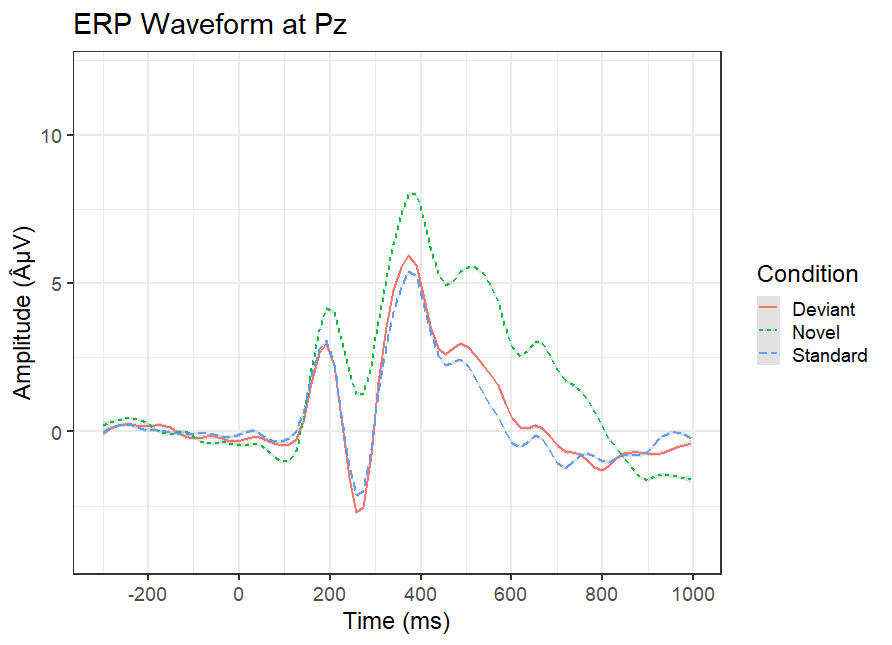

Supplement: Supplementary Figure 2 — ERP waveform for standard, deviant and novel visual oddball stimuli. [file Image_2.PNG]
